# Alleviate environmental concerns with biochar as a container substrate: a review

**DOI:** 10.3389/fpls.2023.1176646

**Published:** 2023-07-27

**Authors:** Ping Yu, Kuan Qin, Genhua Niu, Mengmeng Gu

**Affiliations:** ^1^ Department of Horticulture, University of Georgia, Griffin, GA, United States; ^2^ AgriLife Research Center, Department of Horticultural Sciences, Texas A&M University, Dallas, TX, United States; ^3^ Department of Horticulture and Architecture, Colorado State University, Fort Collins, CO, United States

**Keywords:** peat moss, substrate properties, pathogens, economic benefits, potted plant

## Abstract

Peat moss has desirable properties as a container substrate, however, harvesting it from peatland for greenhouse/nursery production use has disturbed peatland ecosystem and caused numerous environmental concerns. More recently, many nations have taken actions to reduce or ban peat moss production to reach the carbon neutral goal and address the environmental concerns. Also, the overuse of fertilizers and pesticides with peat moss in greenhouse/nursery production adds extra environmental and economic issues. Thus, it is urgent to find a peat moss replacement as a container substrate for greenhouse/nursery production. Biochar, a carbon-rich material with porous structure produced by the thermo-chemical decomposition of biomass in an oxygen-limited or oxygen-depleted atmosphere, has drawn researchers’ attention for the past two decades. Using biochar to replace peat moss as a container substrate for greenhouse/nursery production could provide environmental and economic benefits. Biochar could be derived from various feedstocks that are regenerated faster than peat moss, and biochar possesses price advantages over peat moss when local feedstock is available. Certain types of biochar can provide nutrients, accelerate nutrient adsorption, and suppress certain pathogens, which end up with reduced fertilizer and pesticide usage and leaching. However, among the 36,474 publications on biochar, 1,457 focused on using biochar as a container substrate, and only 68 were used to replace peat moss as a container substrate component. This study provides a review for the environmental and economic concerns associated with peat moss and discussed using biochar as a peat moss alternative to alleviate these concerns.

## 1 Introduction

Peatlands contribute vital ecological services such as storing organic carbon (C) and nitrogen (N), regulating water, influencing methane (CH_4_), and providing habitats (Leifeld and Menichetti, 2018; Humpenöder et al., 2020). Peatlands occupied around 4% of the terrestrial surface but stored 644 Gt of C or 21% of the global total soil organic C stock (Yu et al., 2010; Scharlemann et al., 2014; Dargie et al., 2017; Leifeld and Menichetti, 2018). Northern peatlands alone store 17 Gt N, and for well-grown sphagnum peatlands, one single sphagnum farming site takes up N at 35~56 kg ha^-1^ yr^-1^ (Temmink et al., 2017; Hugelius et al., 2020). By regulating water flows, peatlands help minimize the risk of flooding and drought and prevent seawater intrusion (Rizzuti et al., 2004). In the peatland system, up to 90% of biologically CH_4_ produced is consumed due to activities of methanogens and methanotrophs (Liebner et al., 2011). Peatlands also provide precious habitats for different wild animals (Alexander et al., 2008).

Harvesting peat moss, a commonly used container substrate in horticulture, has caused numerous environmental concerns. Large scale peatlands drainage caused carbon dioxide (CO_2_) and nitrous oxide (N_2_O) emissions more than 2 Gt CO_2_-eq yr^-1^. The CO_2_ emissions from the drained peatlands are estimated at 1.3 Gt CO_2_ annually, which is equivalent to 5.6% of the global anthropogenic CO_2_ emissions (Nature, 2017). In addition, the drainage of peatland, with other gas and fuels extractions, contributed 23% of the total CH_4_ budget of 500 to 600 tera gram per annum (Reumer et al., 2018), and increased the total CH_4_ emissions from 334 Tg yr^-1^ to 366 Tg yr^-1^ (Saunois et al., 2020). Peatland extraction reduced surface and groundwater quality, and increased land compaction (Temmink et al., 2017). Moreover, peat extraction has caused 15% of global peatland habitats lost for wild animals, including Bornean Orangutans (Barthelmes, 2016; Nature, 2017; Vaughn et al., 2018). If the peatland extraction trend continues, the cumulative of greenhouse gases (GHGs) CO_2_ equivalent emission would reach to 249 Gt by 2100 (Heck et al., 2021). Among the 17 United Nations Sustainable Development Goals, 8 goals are closely related to ecosystem interference and global warming (The United Nations Sustainable Development Goals, 2015). Therefore, reducing the use of peat moss and finding a peat moss replacement is necessary and urgent.

**Figure 1:**
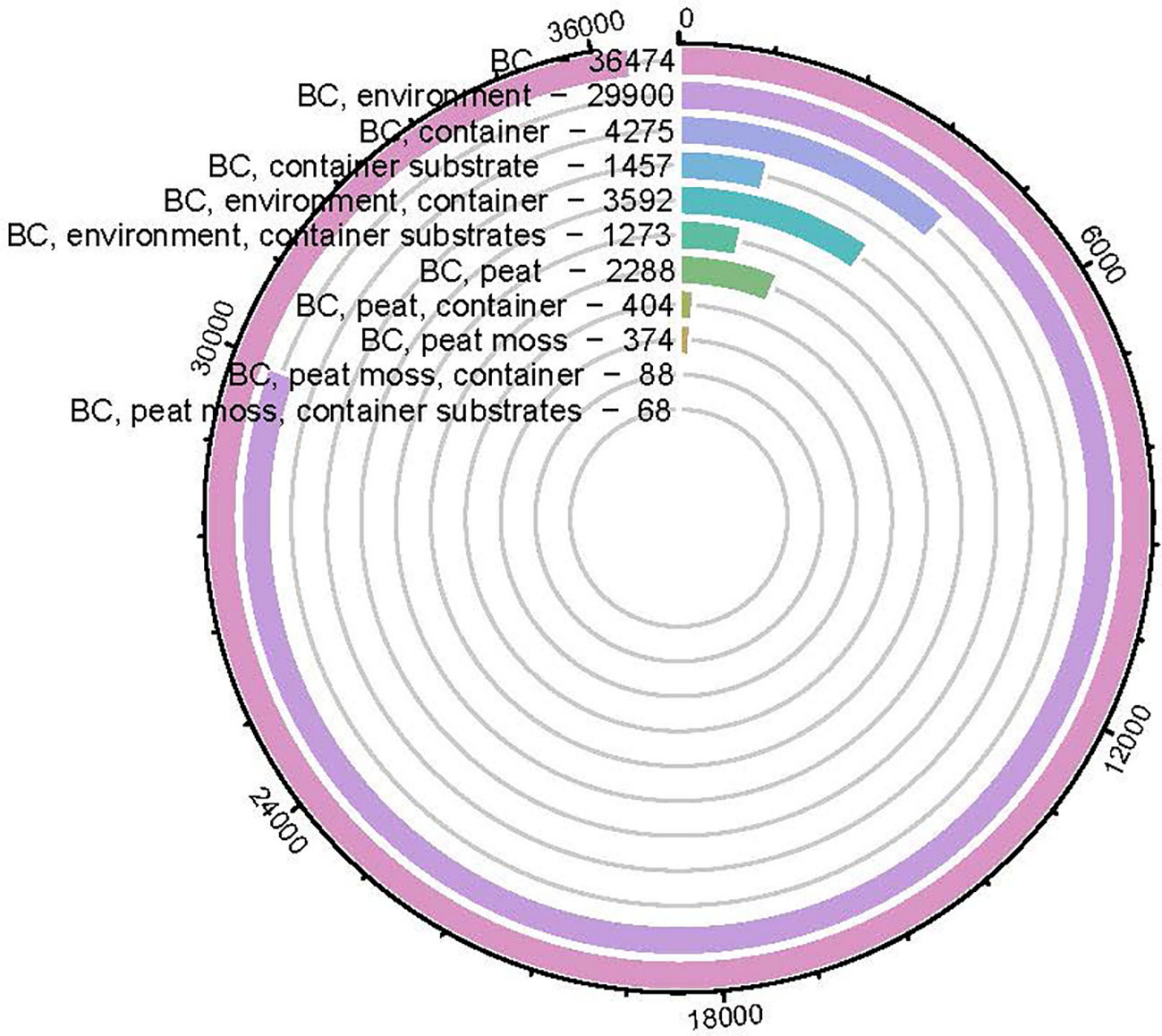
Circular bar-plot indicating the number of biochar (BC)-related articles published from 2010~2023 based on key words searching in Science Direct database.

There are several potential organic materials that can be used as peat moss replacements, including coconut coir, rice hull, and wood bark. In addition to these materials, recently, biochar has received attention as a superb peat moss alternative with many advantages. Since it has been long discovered in the amazon rainforest as terra preta (black soil), biochar has been evaluated and studied from researchers in the past two decades (Denevan and Woods, 2004). Biochar is a carbon-rich material with porous structure produced by the thermo-chemical decomposition of biomass in an oxygen depleted or oxygen-limited atmosphere (Demirbas and Arin, 2002; Lehmann, 2007; Nartey and Zhao, 2014). Data from the literature were obtained from web of science database from 2010 to 2023 with searching terms such as “biochar”, “biochar container substrate”, “biochar environment”, “biochar peat moss” etc. The number of biochar-related publications increased from 76 to 3,6474 in the past two decades (**Figure 1**.), with its main applications being soil amendments (Lehmann et al., 2011), pollutant removal (Ahmad et al., 2014), beneficial bacterial carrier (Belonogova et al., 2018), and mitigate climate change (Woolf et al., 2010). Most of studies were either focused on increasing crop growth or reducing non-peat moss related environmental concerns such as carbon sequestration, contaminants remediation, greenhouse gas emission reduction (Das et al., 2020; Bolan et al., 2022a; Bolan et al., 2022b). There were decent number of studies concentrated on biochar production, characterization and engineering (Albert et al., 2021; Basak et al., 2022; Bolan et al., 2023). Among these 36,474 publications, 1457 focused on using biochar as a container substrate, and only 68 were used to replace peat moss as a container substrate component.

Based on the existing information, using biochar as a container substrate holds immense potential to offer substantial environmental and economic benefits for various compelling reasons. Unlike peat moss, which needs a long time to regenerate, biochar is considered as a renewable material since it can be derived from various and fast generating feedstocks (Yan et al., 2020), ranging from plant-based material such as green waste (Tian et al., 2012), wood (Vaughn et al., 2015; Gascó et al., 2018; Fascella et al., 2020; Ferlito et al., 2020), straw (Spokas et al., 2009; Spokas et al., 2010; Vaughn et al., 2013; Hansen et al., 2015; Hansen et al., 2016), bark (Hina et al., 2010), rice hull (Locke et al., 2013), wheat straw (Vaughn et al., 2013; Xu et al., 2016; Xu et al., 2016) to other sources such as deinking sludge (Méndez et al., 2015; Méndez et al., 2017). For the same reason, biochar has price advantages over peat moss, especially when biochar is made from feedstocks from local industries and farms (Yan et al., 2020). Using biochar as a peat moss replacement protects peatland from further drainage for peat moss harvesting, thus protecting peatland ecosystems and reducing GHGs emissions (Hao et al., 2010; Ro et al., 2010; Cornelissen et al., 2013; Conversa et al., 2015). Moreover, producing straw biochar and adding it into agriculture production can directly reduce CO_2_ emission by 47% and 57% for rice and maize, respectively (18,479.35–37,457.66 kg) and reduce CH_4_ and N_2_O emission (Ji et al., 2018; Xu et al., 2018). Biochar could increase water and nutrient use efficiency, reduce fertilizer and pesticide runoff, render equivalent plant yield, thus providing both environmental and economic benefits (Guo et al., 2018a; Huang et al., 2019a; Yan et al., 2020; Yu et al., 2020a).

As such, this article discussed the use of biochar to replace peat moss as a container substrate to alleviate environmental issues by collecting exponentially increased number of publications and reviewing them to explain how the properties of biochar make it a viable alternative to peat moss, how biochar helps in reducing fertilizer pollution and the leaching of nutrients, how it addresses issues related to peatland disturbance, and how it provides potential economic benefits. This article also provides new insights into the research gap, state-of-the-art challenges of using biochar on a large scale and the possible solutions. The future research directions of using biochar as a peat moss alternative was also discussed. The structures and key points for this study are: 1) biochar has huge potential to replace peat moss as a container substrate component; 2) biochar can provide environmental and economic benefits; 3) more actions need to be taken to use biochar in horticulture area in a large scale.

## 2 Peat moss used as a container substrate

### 2.1 Properties of peat moss

Peat moss has long been widely used a container substrate due to its suitable properties, which allows it to support plants, hold nutrients, retain water, and change gases (Yeager et al., 2007; Nelson, 2012). Despite its suitable properties, peat moss could have rewetting and leaching issues (Gaudig et al., 2017; Kumar, 2017). The drying process during commercial peat moss production made it hydrophobic (Beardsell and Nichols, 1982; Gaudig et al., 2017; Kumar, 2017), and as an organic material, peat moss breaks down during greenhouse practices, which changes its hydrophobicity intensity and causes rewetting issues (Valat et al., 1991; Dekker and Ritsema, 2000). Especially after dried out, when the moisture content decreases below 20%, peat moss requires a longer time to rewet as it becomes more hydrophobic (Michel et al., 2001). Additionally, peat moss-based substrate leads to more nutrient leaching than bark substrate, which may be due to its higher content of macropores (>50 nm, 11%) comparing to bark substrate (7%) (Drzal et al., 1997).

### 2.2 Environmental concerns caused by peat moss

Harvesting peat moss for container substrate from peatland has interfered peatland’s ecological functions (Leifeld and Menichetti, 2018). Peat moss harvesting reduced peatland C capacity, thus hindered its climate change mitigation capacity (Alexander et al., 2008). Also, harvesting peat moss disturbed N and CH_4_ cycles (James et al., 2021). Additionally, peatland disturbance may bring challenges to the native animals, making it harder for them to find new habitats, thus reduce ecosystem biodiversity (Alexander et al., 2008).

Besides interfering with peatland’s ecological functions, peat moss, as a container substrate component, also creates environmental concerns due to nutrient runoff as well as pesticide runoff (Michel et al., 2001; Kumar, 2017). In a common nursery production, a 15% leaching fraction was recommended to prevent the buildup of soluble salts in the container substrate (Cahn and Phillips, 2019). However, extensive irrigation, fertilizers, and pesticides were more often applied to containers to reduce the risk of crop failure (Savvas et al., 2013). Plants can only use 50% of nitrogenous fertilizers applied even under ideal conditions (Sönmez et al., 2008; Savci, 2012). The excessive nitrogen (N), phosphorus (P), and potassium (K) were lost through runoff, causing environmental concerns such as eutrophication, dead zones, and algal blooms (Power and Schepers, 1989; Zhu et al., 2004; Savci, 2012). Because of the low irrigation efficacy (80% of water runoff) in container production, highly soluble pesticides such as acephate, glyphosate, and mefenoxam are likely to dissolve and move with runoff water to a containment water body (Poudyal and Cregg, 2019). A 10-year survey of major streams and groundwater found that 97% of stream water and 61% of shallow groundwater near agricultural areas had one or more pesticides present (Stone et al., 2014).

### 2.3 Challenges of peat moss

Peat moss encounters production challenges as its volume and area have been largely reduced. The total volume and area of global peatlands have been decreased at a rate of 0.05% annually and by 10%~20% since 1800 owing to harvesting and land development (Temmink et al., 2017; Heck et al., 2021). Peat production was estimated to have decreased in 2019 in some peatland-rich countries (Temmink et al., 2017). Peatland area in Estonia has declined from 22% coverage of the country to only 5.5% for the past decade (Orru and Orru, 2006; Karofeld et al., 2020). In Ireland, around 84% of ombrotrophic peatlands (bogs) have been affected by peat extraction (Renou-Wilson et al., 2019). In Germany and Netherlands, 98% and 95% domestic peatland area have degraded due to the extensive peat moss harvesting (Barthelmes, 2016).

Peat moss also faces legislation challenges due to the implementation of peatland restoration projects and carbon neutral plans (Peng et al., 2018). Several European countries including Belarus, Ireland, and Sweden, were planning or implementing peatland restoration projects, reducing peat production across Europe in the future (Carlile and Coules, 2011). In Canada, among the total of 27, 615 ha peat moss production areas, more than 31% has been or is currently restored or reclaimed, with another 3% converted to other land-use (Shotyk, 1988). Also, the UK and Europe have legislated laws in order to protect the peatland from being over harvested (Alexander et al., 2008; Carlile and Coules, 2011). In 2019, Ireland announced its plan to stop all peat harvesting by 2028 (Brioche, 2020). In the same year, Finland announced its goal to become carbon neutral by 2035 by phasing out peat production (Brioche, 2020).

## 3 Biochar replacing peat moss as a container substrate

### 3.1 Biochar has suitable properties

Although biochar properties vary widely, many types of biochar could fall into the recommendation range either by itself or by combining with other components (Huang and Gu, 2019). Detailed biochar properties have been reported by Lan et al. (Huang and Gu, 2019), we summarized in **Table 1** to compare several differences between biochar and peat moss-based substrates used in containers. For the most commonly used container substrate components such as peat moss and perlite, their total porosity was high, 83% and 92%, respectively, indicating low total porosity components need to be included to reach the ideal range (50-85%). As far as pH concerned, peat moss and vermicompost had a low pH lower than 5, 4.3-5 and 4.8, respectively, indicating that other alkaline components such as mixed hardwood biochar (pH 10.8-11.8) need to be incorporated to reach the ideal pH range (5.4-6.5) for container substrate (**Table 1**). For vermicompost and chicken manure, since their electricity conductivity and bulk density were high, 6.7 and 32.9 mS cm^-1^, 0.38 and 0.62 g cm^-3^ respectively, their amount needs to be considered carefully when adding them into container substrates. Pinewood biochar, mixed hardwood biochar, and sugarcane bagasse biochar used in our previous studies had similar total porosity (74~85%), air space (3~34%), and bulk density (0.09~0.17 g cm^-3^) to peat moss (83%, 19%, and 0.08 g cm^-3^, respectively) and peat moss-based commercial substrate (71~78%, 3~20%, and 0.11 g cm^-3^, respectively) (Guo et al., 2018b; Webber et al., 2018; Huang et al., 2019a; Yan et al., 2020; Yu et al., 2020b).

**Table 1:** The physical properties including total porosity (TP, %), container capacity (CC, %), air space (AS, %), bulk density (BD, g cm-3), and particle size (PS, mm); chemical properties including pH, electrical conductivity (EC, mS cm-1), cation exchange capacity (CEC, meq 100g-1) and biological properties (microorganisms, MC) of several types of biochar and peat moss-based commercial substrate from our previous studies.

Properties	TP (%)	CC (%)	AS (%)	BD (g cm^-3^)	PS (mm)	pH	EC (mS cm^-1^)	CEC (meq 100g^-1^)	MC
Ideal Range	50~85	45~65	10~30	0.19~0.7	N/A	5.4~6.5	<0.75 (seedlings) <1.5(general crops)	6~15	N
PB	83	48.6	34.2	0.17	0.59~2	5.4	N/A	N/A	N
HB	85	60.3	24.4	0.15	67.3% >2	10.8~11.8	0.11	N/A	N
SBB	74	66~85	3~9	0.09~0.11	0.17(mean)	5.9	0.08	N/A	N
Peat moss	83	64	18.9	0.08	N/A	4.3-5	N/A	7~13	N
Perlite	92	59	34	0.05	N/A	7.3	0.01	~0	N
VC	75	72	3	0.38	89.4%<2	4.8	6.7	N/A	Y
CM	64	60	4	0.62	89.4%<2	7.5	32.9	N/A	Y
CS1	74~78	58~71	3~20	0.09~0.1	65.2%<2	N/A	N/A	N/A	N
CS2	71~75	84	15	0.11	N/A	6.8	0.07	N/A	N
PCS	79~97	47~85	12~31	0.15	3~6	6.5~6.75	0.18	N/A	N

Based on the studies from (Guo et al., 2018b; Huang et al., 2019a; Peng et al., 2018; Webber et al., 2018; Yan et al., 2020; Yu et al., 2020b). PB, pinewood biochar; HB, mixed hardwood biochar; SBB, sugarcane bagasse biochar; VC, vermicompost; CM, chicken manure; CS1, peat moss-based commercial substrate for plants growing; CS2, peat moss-based commercial substrate for plants propagation; PCS, pine bark-based commercial substrate; N/A, not applicable; N/Y in the microorganism column means mixes do not contain/contain microorganisms.

Unlike peat moss, which may encounter rewetting difficulties, certain types of biochar used in containers are easy to rewet due to its larger surface areas and pore size distribution (Lehmann et al, 2011). Biochar made from organic materials at 400 ~1,200°C, has larger surface area than peat moss because its higher micropores content (Lee et al., 2015). The surface area of biochar increased because high temperatures changed more macropores into mesopores/micropores in biochar (Lee et al., 2015). Micropores contributed largely to biochar surface area, endowing high adsorptive capabilities on the biochar and allowing small dimension molecules, such as gases and solvents to be absorbed (Lehmann et al, 2011). Thus, when the same irrigation practice applied, biochar would encounter less difficulties in rewetting than peat moss or peat moss-based substrate (Drzal et al., 1997).

### 3.2 Biochar has benefits on nutrients supply and absorption

Biochar was proposed to be beneficial to plant nutrient absorption because it could provide nutrient resources depending on its feedstock and production method. Lin et al. mentioned that acacia saligna biochar produced from at 380°C and sawdust at 450°C contained 17.7 and 16.2% of humics (humic-like and fluvic-like materials), which can serve as biostimulant and be assimilated by plants (Lin et al., 2012; Ding et al., 2016). Similarly, biochar made from gasified rice hulls at 815 ~ 871°C could be used as P and K fertilizers as the 5.4 g (0.19 oz) biochar sample released 35.2 mg (0.0012 oz) P and 50.1 mg (0.0018 oz) K in water solution for container crops over a short production cycle of 6 weeks (Altland and Locke, 2013). Pine bark biochar produced from 450°C fast pyrolysis increased mint growth due to its high K and P contents (Yan et al., 2020).

Also, biochar benefits plant nutrient due to its various properties. Adding green waste biochar to the substrate decreased the available N, resulting from biochar’s porous structure induced N binding effects (Altland and Locke, 2012; Tian et al., 2012). Applying sugarcane bagasse biochar or mix hardwood biochar (pH 5.4 and 10.1 respectively) could adjust the substrate pH to around 6~8 (Yu et al., 2020b). The suitable substrate pH range (6~8) could promote K content, causing Mg and Ca deficiency due to the antagonism and/or synergism relationships among nutrients (Landis, 2005; Taiz and Zeiger, 2010).

### 3.3 Biochar effects on plant diseases

Soil-borne diseases affect potted plants’ marketability and are hard to control (Katan, 1997; Graber et al., 2014; Puertolas et al., 2018). There are 10~20% of attainable crop yields loss caused by soil-borne diseases and the economic losses in USA are more than $4 billion (Graber et al., 2014). Soil-borne diseases control becomes more challenging due to trade globalization (Daughtrey and Benson, 2005; Puertolas et al., 2018). For instance, *Phytophthora ramorum* has survived for eight months in root balls and potting substrates of rhododendron plants, affecting the plants marketability worldwide (Appiah et al, 2004; Vercauteren et al., 2013). *Fusarium oxysporum f. sp papaveris*, a fungi pathogen attacking Papaveraceae plants, largely affected Papaveraceae plants marketability in Italy (Bertetti et al., 2018).

**Figure 2:**
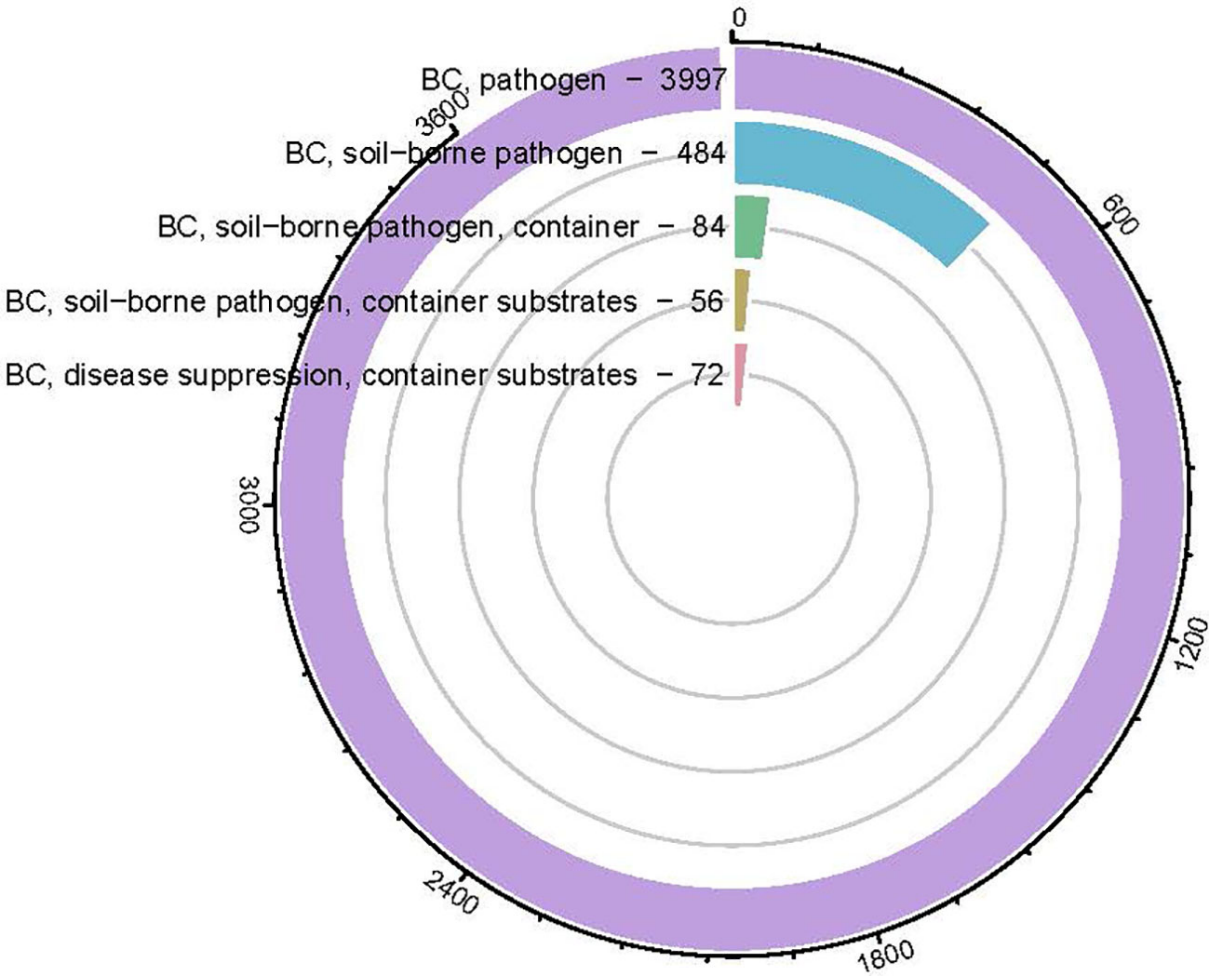
Circular bar-plot indicating the number of biochar (BC) pathogen-related articles published from 2010~2023 based on key words searching in Science Direct data base.

As a container substrate to replace peat moss, the effects of biochar on soil-borne pathogen has been less reported than that of plant growth, which had positive, neutral, and negative effects (Huang and Gu, 2019; Yu et al, 2020b). To date, there aren’t enough studies about the biochar effect on plant health (**Figure 2**.), based on the Scientific Report database, among the 36,474 biochar publications (**Figure 1**.), only 3,997 were pathogen related, less than 11%. The majority of those pathogen studies were conducted in field, only 84 were conducted in containers. The dose of biochar is relatively low (ranging in most cases between 0.5~5%, **Table 2**) and most of the studies were conducted on edible crops such as tomato, pepper, strawberry, asparagus, lettuce, cucumber, beans etc (Copley et al., 2015; Mehari et al., 2015; Caroline et al., 2016; Jaiswal et al., 2017). The highest dose of biochar used in those studies is testing balsam fir bark and spruce bark biochar (475°C) on *Pythium ultimum* on sweet pepper, lettuce, basil, geranium and coriander at 50% (Gravel et al., 2013). Among those studies in **Table 2**, there were only two studies tested biochar effects on disease for ornamental crops which was red maple, red oak and geranium (Zwart and Kim, 2012; Gravel et al., 2013).

**Table 2:** Biochar effects on plant pathogens.

Host plants	Pathogen	Biochar feedstock	Biochar temperature °C	Biochar rate		Reference
bean	*Rhizoctonia solani*	eucalyptus wood, greenhouse wastes	350, 600	0,1%, 3% (w/w)		(Copley et al., 2015)
cucumber, tomato, lettuce, sweet pepper etc.	*Rhizoctonia solani*	maple bark biochar		0,1%,3%,5% (w/w)		(Elmer and Pignatello, 2011)
strawberry	*Botrytis cinerea, Colletotrichum acutatum and* *Podosphaera apahanis*	citrus wood (CW), greenhouse wastes (GWC)	GWC at 450	1% or 3% (w/w)		(Harel et al., 2012)
asparagus	*Fusarium oxysporum f.* sp. *asparagi;* *F. proliferatum (fusarium crown and root rot)*	hardwood dust charcoal	N/A	0,1.5%,3% (w/w)		(Jaiswal et al., 2015)
asparagus	*Fusarium oxysporum f.* sp. *asparagi (Fusarium root rot)*	coconut fiber charcoal	N/A	0,10%,30% (v/v)		(Graber et al., 2014)
tomato	*Ralstonia solanacearum (bacterial wilt)*	municipal bio-waste charcoal, wood charcoal	N/A	0, 20% and other not-specified concentrations (v/v)		(Nerome et al., 2005)
red oak and red maple	*Phytophthora cinnamomi and P. cactorum (stem canker)*	pine	Between 550 and 600	0, 5, 10 and 20% (v/v)		(Zwart and Kim, 2012)
sweet pepper, lettuce, basil, geranium and coriander	*Pythium ultimum*	balsam fir bark and spruce bark	475	50% (v/v)		(Gravel et al., 2013)
tomato	*Fusarium* spp.	eucalyptus wood pepper plant waste	350/600	0,0.5%,1%,3%(w/w)		(Jaiswal et al., 2017)
tomato pepper	*Botrytis cinereal* *Leveillula taurica*	citrus wood	N/A	1%,3%,5%(w/w)		(Elad et al., 2010)
lettuce	*OTC (antibiotic)*	bamboo	600	2%		(Duan et al., 2017)
cucumber	*Rhizoctonia solani*	eucalyptus wood and greenhouse wastes	350/600	0%~3%		(Jaiswal et al., 2014)
beans	*Rhizoctonia solani*	eucalyptus wood and greenhouse wastes	350/600	0%~3%		(Jaiswal et al., 2015)
rice	*Meloidogyne graminicola*	holm oak wood	650	0.6%, 1.2%, 2.5%, 5.0%		(Huang et al., 2015)
tomato	*Botrytis cinerea*	greenhouse wastes	450	0, 1, and 3%(w/w)		(Mehari et al., 2015)
lettuce strawberry	*Rhizoctonia solani* *Botrytis cinerea*	holm oak wood	650	0, 1, and 3% (w/w)		(Caroline et al., 2016)
carrot	*Pratylenchus penetrans*	pinewood, pine bark, wood pellets, spelt husks	500	0.80%, 0.92%, 1.24%, 0.64%		(George et al., 2016)
sweet pepper, tomato, lettuce, carrot, radish	*Rhizoctonia solani*	maple wood bark	700	0,1%,3%,5% (w/w)		(Copley et al., 2015)

N/A, Not applicable.

Similar to its effects on plant growth, biochar effects on plant health vary depending on plant species, biochar rates and types (Frenkel et al., 2017). Gravel et al. (2013) found that adding 50% of balsam fir/spruce bark biochar caused higher pathogen root colonization rate in all other crops except for coriander. Adding 30% coconut biochar increased plant health (Graber et al., 2014). Kadota and Niimi claimed that maple bark biochar improved the quality of several plant species, shortened the number of days needed for flowering, and increased plants survival rates (Kadota and Niimi, 2004). Adding 3% (w/w) wood-derived biochar with pre-conditioning such as pre-planting fertigation of the media reduced pre-emergence damping off caused by *Pythium aphanidermatum* by 71% for cucumber seedlings (Jaiswal et al., 2019). Incorporating biochar at rates of 10-30% (by vol.) increased strawberry fresh weight by 5-10% and reduced *Phytophthora* presents (Blok et al., 2019). Earthworm, microalgae biomass and 6% biochar mix increased tomato, pepper and eggplant seeds’ resistance for *Pythium* sp., increased germination rate by 34% (Alshehrei et al., 2021). Adding 20% and 50% of mixed hardwood biochar decreased poinsettia root rot disease caused *Pythium aphanidermatum* and pepper blight disease caused by *Phytophthora capsica*, respectively (Yu et al., 2021; Yu et al., 2023).

The potential mechanisms on how biochar may influence plant disease include both direct and indirect influence on pathogen: 1) biochars’ chemical compounds affect pathogen growth; 2) biochars’ physicochemical properties improve soil nutrients availability and abiotic conditions; 3) biochars’ physical properties help absorb toxins and enzymes produced by pathogens, reducing virulence; 4) biochars’ presence induces systemic resistance in host plants; 5) biochars’ physical properties enhance abundance and/or activities of beneficial microbes; 6) biochar induced disease suppression related gene expression (Graber et al., 2014; Bonanomi et al., 2015; Jaiswal et al., 2018; Jaiswal et al., 2020; Rasool et al., 2021; Ji et al., 2022; Liu et al., 2022).

## 4 Environmental benefits of biochar as a container substrate

### 4.1 Biochar protects peatland

The horticulture industry demands a large amount of peat moss as container substrates. Around 0.15 M m^3^ of peat moss were used in container plants production, accounting for 86.5% of the total imported peat moss in the United States (USDA-NASS, 2018). In the United Kingdom, 0.06 M m^3^ peat moss were used in horticulture, including container plants, bedding plants, vegetables, soft fruit, and cut flower production. In Europe, around 2.6 M m^3^ peat moss were used in horticulture, with the total ratio of peat in media for plant growth being 99% in Estonia, 99% in Lithuania, 92% in Latvia, 88% in Finland, 87% in Ireland, 87% in Denmark, 87 in Sweden, and 81% in Germany (Kitir et al., 2018).

Replacing peat moss with biochar protects peatland from further disturbance. The highest rate for biochar replacing peat moss as a container substrate is 80% with pine bark biochar (Guo et al., 2018b; Huang et al., 2019b). If 80% of peat moss can be replaced by pine bark biochar, 0.12 M m^3^, 0.05 M m^3^ and 2.08 M m^3^ peat moss can be saved annually in the United States, in the United Kingdom, and in Europe, respectively. Global average dry biomass Sphagnum production is around 260 g m^-2^ yr^-1^, depending on species and locations (Gunnarsson, 2005). Considering the commercial peat moss bulk density is 0.1 g cm^-3^, if 80% of peat moss substrate can be replaced by pine bark biochar, 46.2 M m^2^, 19.2 M m^2^, and 800 M m^2^ of peatland can be saved annually from being disturbed for the United States, the United Kingdom, and Europe, respectively.

### 4.2 Biochar reduces chemical leaching

#### 4.2.1 Biochar reduces nutrient leaching

As aforementioned, fertilizer tends to be over-used in greenhouse/nursery production and plants can only use 50% of fertilizers applied (Sönmez et al., 2008; Savci, 2012). The rest of the other half of fertilizers were either lost in evaporation and/or reactions with organic compounds (Savci, 2012). Moreover, since the majority of fertilizers haven’t been absorbed by plants, they can reach ground water and contaminate ground water (Power and Schepers, 1989; Zhu et al., 2004).

Biochar replacing peat moss as a container substrate reduces nutrient runoff either by providing additional nutrient content or alternating substrates’ properties. Adding 15-20% gasified rice hull biochar (815 ~871 °C) in a peat-based substrate reduced nutrients such as NH_4_^+^, NO_3_^-^, H_2_PO_4_^-^, HPO_4_^2-^, and K^+^ leaching as it provided sufficient potassium (K) amount for geranium and tomato plants growing in containers (Altland and Locke, 2017). Jahromi et al. (2018) found that switchgrass (1,000 °C) biochar-amended substrates reduced the total nutrients lost from hydrangea containers because biochar addition increased substrate water holding capacity. Altland and Locke (2013) demonstrated that adding 10% saw dust biochar to peat moss-based substrate increased nitrate and phosphate retention and subsequently reduced their leaching. Adding conifers wood biochar (500 °C) into container substrate for lavender production reduced K leaching as it increased K content of the growing substrates significantly (Fascella et al., 2020). Woodchip biochar (450~600 °C) decreased more extractable total N including NO_3_-N than peat moss substrates with similar seedlings growth (Prasad et al., 2018). Similarly, adding forest wood biochar (700 °C) at 7.5% with additional fertilizer reduced NO_3_-N, K and P leaching compared to the peat substrate. Adding fresh wood screening at 7.5% and 15% (500-600 °C) decreased NH_4_-N and K leaching compared to the peat substrate under both 1-fold and 1.5-fold fertilizer conditions (Chrysargyris et al., 2019).

#### 4.2.2 Biochar decreases pesticides usage and leaching

The over-use of pesticides in greenhouse production also caused environmental concerns (Ayoub, 1999; Bolognesi, 2003). In the United States, among the total usage of pesticide, around 90% of pesticide comes from agricultural production (Atwood and Paisley-Jones, 2017). Pesticides contaminate the environment via surface runoff, spray drift, and subsurface flow, which is the major pathway for pesticides entering water bodies (Zhang et al., 2018). Leaching can rapidly transport pesticides to surface and subsurface receiving waters (Roseth and Haarstad, 2010). The best management practices are recommended for nurseries to reduce pesticide contamination, yet, the best management practices alone may not completely remove pesticides contamination (Grant et al., 2019).

Biochar has been reported as a good sorbent for efficient removal of chemicals, and its efficacy depends on many factors including biochar types, effect of time, adsorbent dosage, chemical concentration and pH. Taha et al. (2014) demonstrated that biochar made from corn stover and rice straw adsorbed many types of pesticides including organophosphates (diazinon and malathion) and neonicotinoids (imidacloprid and acetamiprid). Mandal et al. (2017) reported that rice straw biochar had the highest adsorption rate for atrazine and imidacloprid. Baharum et al. (2020) found that activated coconut fiber biochar (700°C) removed 98.96% and 87.93% of diazinon respectively when modified with phosphorus acid and sodium hydroxide at pH 7. Ponnam et al. (2020) described that biochar produced from the neem tree bark (300°C) provided a 95.2% desirability on removal Bentazone with response (adsorption uptake) of 79.40 mg/g, for initial concentration of insecticide (50 mg/L), adsorbent dosage (0.448 g), time 30.0 min and pH 2. Gámiz et al. (2019) demonstrated that aged oak wood biochar (550°C) had a significantly higher removal rate (>85%) of three highly persistent and ionizable pesticides (imazamox, picloram, terbuthylazine) than the fresh biochar (<16%).

## 5 Economic benefits of biochar as a container substrate

Biochar provides large potential economic values as the market of biochar and biochar supply companies are growing. According to the transparency market research (Doe, 2014; Natural-Resources, 2017), the evaluated worth of global biochar market reached $0.44 M in 2016, and it is expected to experience a Compound Annual Growth Rate of 14.5% from 2017 to 2025 and reach a valuation of $1.48 M by 2025. Also, the number of biochar supply companies increased. There were approximately 150 biochar supply companies in 2013, mostly of them were small garden and specialty retailers, however, the number of biochar companies doubled in 2015 (Cedergreen et al., 2009; Jirka and Tomlinson, 2015).

### 5.1 Biochar decreases peatland restoration costs

Peatland restoration requires high economic costs such as techniques costs, rewetting and recurring costs, as well as maintenance costs (Glenk and Martin-Ortega, 2018; Humpenöder et al., 2020; Karofeld et al., 2020). The costs associated with restoration range from $280 ha^-1^ to $14,016 ha^-1^ (Moxey and Moran, 2014). A one-time cost of $7,000 ha^-1^ for initial rewetting and recurring was estimated, with another cost of $200 ha^-1^ yr^-1^ maintenance and/or $140 ha^-1^ yr^-1^ management costs (Glenk and Martin-Ortega, 2018).

Replacing peat moss with biochar as a container substrate largely reduces peatland restoration costs because biochar production does not degrade the peatland ecosystem. With around 10.3 M ha peatland area needs to be restored (Humpenöder et al., 2020), an estimated $72.1 billion one-time rewetting and recurring costs with another $2.06 billion and/or $1.44 billion maintenance and management costs could be saved annually by replacing peat moss with biochar.

### 5.2 Biochar reduces substrate costs

Replacing peat moss with biochar as a container substrate can bring large economic benefits due to its potential low price and large demand. The average customer price for sphagnum peat increased from $ 22 m^-3^ in 1986 to $172 m^-3^ in 2018 (Yu et al., 1990; Bwi, 2018). Customers may have to pay higher prices based on the distributors they chose, for instance, the price of peat moss in Greenhouse Megastore is $ 310.7 m^-3^ (Megastore, 2019). Comparing to peat moss, however, the average biochar price is $100 m^-3^, half the price of peat moss from BWI, and one third the price of peat moss from Megastore. Aforementioned, 0.15 M m^-3^, 0.057 M m^-3^, and 2.6 M m^-3^ of peat moss were used in horticulture in the United States, United Kingdom, and Europe, respectively (Kitir et al., 2018; USDA-NASS, 2018). With 80% of biochar being able to replace peat moss as a container substrate (Guo et al., 2018b; Huang et al., 2019b), $8.64 M, $3.6 M, and $149.76 M can be saved annually in the United States, United Kingdom, and Europe, respectively if consumers get peat moss from a cheaper distributor. If consumers get peat moss from a more expensive distributor, $25.2 M, $10.5 M, $436.8 M can be saved annually in the United States, United Kingdom, and Europe, respectively. The actual economic benefits of using biochar to replace peat moss as a container substrate could be even larger if biochar were produced locally, which may lead to an even lower price than the average.

Also, using biochar to replace peat moss as a container substrate brings large economic benefits due to several reasons (**Table 3**). Firstly, peat moss needs a specific condition to growth such as waterlogged, acidic and anaerobic areas while biochar material can be grown anywhere. Secondly, peat moss regrowth rate ranges from 30-40% while biochar materials can reach to100%. Moreover, the price for commercially available peat moss is around $172 m^-3^, if been purchased from wholesale such as BWI, 72% higher than that of biochar. Additionally, peat moss can only be harvested when the depth is more than 2m while biochar materials can be harvest or collected anytime. Peat requires thousands of years to be generated, making it a unrenewable resource (Hugron et al., 2013). With the restoration practices, the average rate of peat moss vertical growth was around 1 mm year^-1^ in the peatland (Savichev et al., 2020). If no restoration practices are launched, the spontaneous revegetation of abandoned peatlands will take even longer (Karofeld et al., 2020). The best suggested harvesting depth for peat moss is 0.25 m from the top soil, meaning after harvesting, peatland needs 25 years or even longer to be able to harvest again (Savichev et al., 2020). The 25 years are more than enough to grow pine trees to merchantable size for biochar production (Butler et al., 2017; Guo et al., 2018b). If we grow other biomass such as sugarcane (or other herbs), miscanthus, and shrubs, the generation of biochar can be 25 times faster than peat moss, providing 25 times the economic benefits of peat moss (Webber et al., 2018; Roy et al., 2020).

**Table 3:** The comparison between peat moss and biochar.

	Peat moss	Biochar
Source	Bog plants: moss, sedge…	Any biomass: sugarcane, bark, municipal wastes…
Formation	Plant material not fully decay	Chemical thermal reaction
Condition	Waterlogged, acidic, anaerobic	Oxygen-free, high temperature
Rate of regeneration	0.5~1mm year^-1^ (naturally)	Comparable to generation of biomass
Renewable	Yes	Yes
Regrowth	Yes, 30~40%	Yes, 100%
Main application	Fuel, soil amendments, potting mix	Fuel, soil amendments, potting mix, pollutant filtration
Price	~$172 m^-3^	~$100 m^-3^
Commercialization	Yes	Limited
Harvesting condition	Depth >2m	N/A
Reclaim rate	~25 yr (harvest wisely)	N/A
Restoration rate	1.5~10 cm year^-1^	N/A

Information based on studies from (Yu et al., 1990; Bwi, 2018; Webber et al., 2018; Karofeld et al., 2020; Savichev et al., 2020). N/A means not applicable.

### 5.3 Biochar reduces chemical costs

Chemical costs in agriculture are high due to large demands and high prices. Global fertilizer demands were projected to 208 M tons with the United States consuming 22 M tons in 2015 at an average price $719 ton^-1^ (Baanante and I.F.P.R. Insitute, 1996; Schnitkey, 2017; EPA, 2019). Global pesticides use in agriculture was 4.12 M tons with USA using 408,000 tons, with the trade reached approximately 5.9 M tons valuing $37.6 billion in 2018 (FAO, 2020). The United States was the top five countries for pesticides imports with trade values ranging $1.4~3.0 billion in 2018 (Wanner et al, 2020).

Replacing peat moss with biochar as a container substrate significantly reduces chemical costs by adding extra nutrients, increasing nutrient use efficiency, and reducing disease incidence. Biochar produced from nutrient-rich raw materials could serve as a source of P and K, reducing the total amount of fertilizer needed for plant growth (Huang et al., 2019a). If using biochar could increase nutrient use efficiency by 50% (Jahromi et al., 2018), $7.91 billion can be saved in the United States, and $74.78 billion worldwide (assuming the average price was $719 ton^-1^) (EPA, 2019). Also, mixed hardwood biochar used in our previous study could reduce 25% disease incidence, leading to less pesticide consumption (Unpublished Data). If using biochar could reduce pesticide usage by 25%, $9.4 billion could be saved globally.

### 5.4 Biochar decrease agricultural waste handling costs

Large amounts of agricultural waste contributed to high waste handling costs. Around 3.9 billion tons of waste were generated annually worldwide with 2.01 billion tons (expected to grow to 3.4 billion tons by 2050) being municipal solid waste (North America contributed 289 M tons) (Kaza et al, 2018). The operating costs for integrated municipal solid waste management, including collection, transport, treatment, and disposal, generally exceed $100 ton^-1^ yr^-1^ (USDA-EPA, 1997).

Using biochar to replace peat moss as a container substrate could significantly reduce agricultural waste handling costs. With pyrolysis for bio-oil purposes, the yield of biochar is from 20%~47% (Ok et al., 2015) (taking the average as 30%). To produce enough biochar for the horticulture industry in USA alone (0.15 M m^3^), assuming all the wastes have similar density as municipal waste, 350 kg m^-3^(USDA, 2008), nearly 0.18 M tons of agricultural waste can be converted, saving $18 M yr^-1^. Similarly, to produce enough biochar for horticulture industry in United Kingdom (0.057 M m^3^), and Europe (2.6 M m^3^), 0.67 M tons, 3.03 M tons of agricultural waste can be converted, respectively, saving millions of dollars on agricultural waste handling.

## 6 Limitations and possible solutions for biochar as a container substrate

Using biochar as a replacement for peat moss as a container substrate provides many benefits, yet it has several limitations. Biochar limitations are mainly from the varied properties and potential toxic substances it may contain, the non-continuous biochar supply-demand loop, and the lack of awareness and production practice of using it as container substrates (Huang and Gu, 2019). Although the number of biochar literature has increased dramatically, there is still little awareness of biochar application among modern farmers (Wu et al., 2017). These limitations may be addressed by providing finically and nonfinancial policy support to motivate business practice change, improving biochar commercial availability, to educate consumers, extending biochar demand, and to establish good production and application practice, exploring more biochar application options (Pourhashem et al., 2019).

### 6.1 Biochar various properties and production

Unlike the well-established sphagnum peat moss, biochar properties vary widely depending on feedstocks, production temperature, and pre- and post-treatment, bringing application difficulties for consumers (Huang and Gu, 2019). Biochar may contain potential toxic compounds such as heavy metals, polycyclic aromatic hydrocarbons (PAHs) and dioxin depending on the raw material and producing conditions (Shackley et al., 2010). When incorporating biochar with heavy metals, PAHs and dioxin into container substrates, plant growth could be decreased.

Biochar’s various properties could be addressed by implementing standard production practices such as using the same feedstock and temperature every time. Currently, most biochar is produced as a by-product from bio-oil-focused process, leading to various properties and toxic compounds (Huang and Gu, 2019; Yu et al., 2020a). Also, biochar made from feedstocks containing toxic compounds, either heavy metal, PAHs or chlorine could contain toxic compounds (Huang and Gu, 2019). As such, businesses can avoid producing toxic containing biochar by selecting feedstock material cautiously. Additionally, biochar various properties can be adjusted to an ideal range for container plants growth by incorporating other components such as bark, perlite, and peat (Guo et al., 2018b).

### 6.2 Biochar non-continuous supply-demand loop

Biochar supply and demand have not created a full loop for the industry yet. Consumers are reluctant to switch from peat moss to biochar due to their lack of awareness and poor biochar availability. Because of the unawareness of using biochar as container substrates, consumers tend to use the well-established and well-supplied peat moss as a major container substrate component, lowering biochar demand. In return, the low biochar demand discourages biochar producing companies due to the low financial benefits. Currently, there are only around 300 biochar companies worldwide, and most of them are small-scale companies, not being able to supply commercial biochar sustainably (Jirka and Tomlinson, 2015). Also, due to the lack of financial motivation, companies are not able to invest in biochar facilities, producing large-scale of container substrate-targeted/grade biochar (Pourhashem et al., 2019).

The non-continuous biochar supply-demand loop can be addressed by establishing related policies to encourage capital investment, providing technology support to reduce the initial production costs (Pourhashem et al., 2019). Academic world needs to pay more attention to the profitability of biochar application in their work (Maroušek et al., 2019). Also, non-financial programs, including extension programs can help educate consumers on biochar economic and environmental benefits and biochar application practices, increasing biochar demand. Additionally, more funding needs to be assigned to biochar research and development programs, exploring more biochar application options to enlarge biochar market margin.

## 7 Conclusions

**Figure 3:**
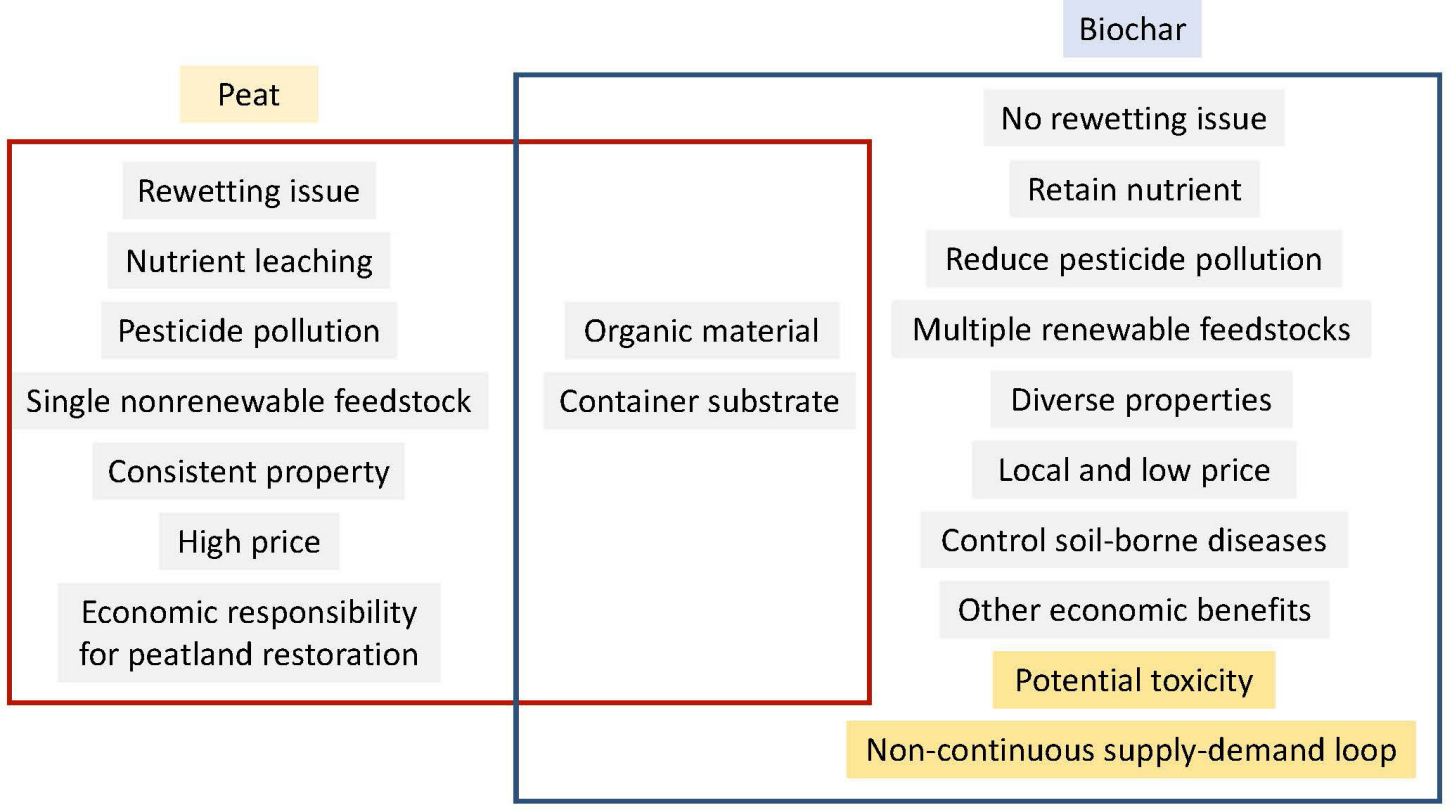
Figure of synthesis of peat moss and biochar comparison.

As summarized in **Figure 3**, using biochar to replace peat moss as a container substrate for plant production provides an environmentally friendly way to address the environmental concerns associated with peatland mining and drainage, and additionally yields multiple benefits. Switching peat moss to biochar as a container substrate for plant production protects peatland ecosystem, increases water and fertilizer use efficiency, reduces greenhouse gas emission, and brings economic benefits. However, to reach biochar’s full potential, biochar limitations such as the lack of awareness, potential toxic compounds, and the non-continuous supply-demand loop need to be addressed soon by establishing both financial and non-financial supports from governments, companies, and research agencies.

Specifically, many container studies have been published on using biochar as an alternative for peat moss, however, most of the studies focused on crop production and the effect of biochar on disease control needs to be explored more. Studies testing the effect of the combination of bio-stimulants and biochar need to be explored for horticulture production. More molecular and physiology studies need to be included to enhance biochar application in horticulture. Also, nano-form of biochar products need to be developed and explored in horticulture. With many studies concentrated on edible crops, testing different biochar sources especially materials that may contain heavy metals such as sewage sledge and municipal waste is essential for safe food production. The facilities for biochar production needs high initial cost, preventing many companies from investing in biochar production, thus, appropriate technology for small to medium sized companies needs to be developed. Furthermore, the appropriate protocols that has been tested need to be shared to establish a uniform guideline for biochar production. Additionally, standardized biochar substrate mixes need to be commercialized for sustainable horticulture production. available, specifically for peat moss alternative growing substrate. In conclusion, using biochar in horticulture as a peat moss alternative can benefit environment economy significantly.

## Author contributions

PY conducted the literature searching, collected, and analysed the data, and wrote the manuscript with the assistance of KQ, GN, and MG.
